# Effect of Calcium on the Setting Time and Mechanical Property of a Red Mud–Blast Furnace Slag-Based Geopolymer

**DOI:** 10.3390/ma17174409

**Published:** 2024-09-06

**Authors:** Yuxiang Chen, Shengping Wu, Hanhui Huang, Feng Rao, Lang Yang

**Affiliations:** 1 Zijin School of Geology and Mining, Fuzhou University, Fuzhou 350108, China; 221627030@fzu.edu.cn (Y.C.);; 2 Fujian Provincial Key Laboratory of Green Extraction and High-Value Utilization of New Energy Metals, Fuzhou 350108, China; 3 School of Engineering, Fujian Jiangxia University, Fuzhou 350108, China; 4 Department of Civil Engineering, Fujian Chuanzheng Communications College, Fuzhou 350007, China

**Keywords:** red mud geopolymer, blast furnace slag, setting time, compressive strength

## Abstract

This study aims to compare the effects of three calcium compounds on the workability, setting time and mechanical properties of red mud (RM)–blast furnace slag (BFS)-based geopolymers. The crystalline phase, hydration process and microstructure of RM-BFS-based geopolymers were characterized by X-ray diffraction (XRD), heat evolution, X-ray photoelectron spectroscopy (XPS), and scanning electron microscope (SEM) tests. The results showed that an appropriate amount of calcium compounds can improve the flowability and compressive strength of the geopolymers, but the excessiveness causes a decrease in strength due to rapid hardening. Other than calcium carbonate, both calcium oxide and calcium chloride played important roles in accelerating the setting times of RM-BFS-based geopolymers. The acceleration in the setting times of geopolymers could be attributed to the calcium hydroxide produced by the dissolution of the calcium compounds, which also provides nucleation sites for the geopolymerization reaction. This study gives new insights into the effect of calcium on the setting times and mechanical properties of geopolymers in the geopolymerization process.

## 1. Introduction

Geopolymers are a class of inorganic cementitious materials with a 3D framework structure, formed by the alkaline activation of aluminosilicate precursors at a certain temperature [1]. The geopolymerization reactions can be categorized into high-calcium systems and low-calcium systems based on the calcium content [2]. In the preparation of geopolymers, calcium-silica-rich raw materials, such as slag, condense quickly and have a certain strength at room temperature [3]. In contrast, silica-aluminum-rich raw materials, such as metakaolin and fly ash, need to be at a certain conditioning temperature to obtain high strength [4,5]. Temuujin et al. [6] showed that calcium compounds improved the compressive strength cured at ambient temperature but reduced those of geopolymers cured at elevated temperature. Yip et al. [7] also proposed that soluble calcium ions can greatly accelerate the hardening progress. Bahmani and Mostofinejad [8] showed that the slag-based geopolymers activated with 10% calcium oxide recorded the compressive strength of 114 MPa and the highest-intensity C-S-H peak and Si-O-T functional band. Thus, the calcium in the raw materials used to prepare geopolymers affects the mechanical properties and reaction products. However, many studies focus on the effect of calcium on the mechanical properties of geopolymers rather than the condensation process such as setting time.

Some studies have shown that the setting times of geopolymers could be influenced by the raw materials [9,10] and activators [11]. Nath and Sarker [12] showed that the addition of slag at 10%, 20% and 30% reduced the initial setting time for fly ash geopolymers from more than 24 h to 290, 94 and 41 min, respectively. Muhammad et al. [13] investigated the optimum mix design of fly ash-based geopolymers, given that the compressive strength increased significantly and the setting times reduced sharply with the increase in ground granulated blast furnace slag (GGBFS) content. Tian et al. [14] produced alkali-activated slag/red mud geopolymers, showing that the setting times of the pastes with 40% to 80% red mud content were appropriate for engineering, with the initial setting time being about 50 min. Xie et al. [15] showed that increasing metakaolin content from 30% to 50% in a slag-based geopolymer delayed both the initial setting time from about 33 to 62 min and the final setting time from 41 to 72 min. These studies have tested the setting time of the geopolymer pastes to investigate the workability of geopolymers, but they seldom systematically studied the mechanism in the condensation process of geopolymers, especially the impact of calcium on reducing the setting time. Recently, several studies used red mud (RM) that might contain calcium for the preparation of geopolymers [16,17,18,19]. Nonetheless, few studies investigate the setting time and mechanical properties of geopolymers using red mud [20].

In this study, red mud (RM) and blast furnace slag (BFS) are used for the preparation of geopolymers. This study aims to investigate the effect of different calcium compounds on the setting time and mechanical properties of RM-BFS-based geopolymers. And three calcium compounds, namely CaO, CaCl_2_, and CaCO_3_, are added into the raw materials in order to investigate the effect of calcium compounds on setting time and mechanical properties of geopolymers. It may give clues to the influence of calcium on the setting time and the workability of geopolymer and may further refine the mechanism of strength development and gel evolution of calcium in geopolymer.

## 2. Materials and Methods

### 2.1. Materials

**Figure 1:**
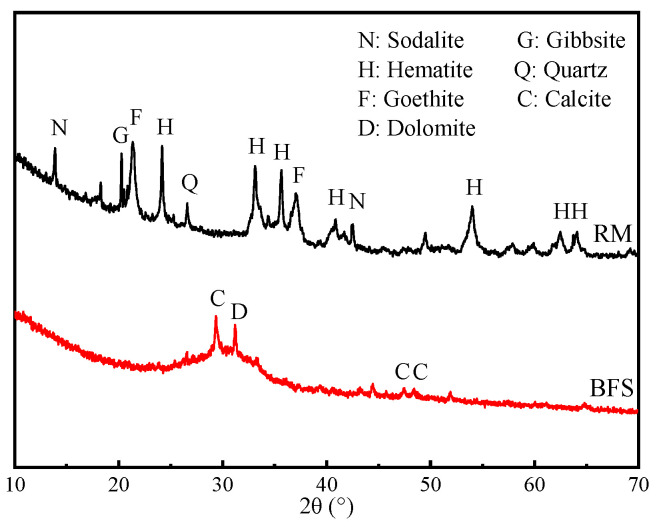
XRD patterns of RM and BFS.

Red mud (RM) was collected from Xinfa Huayu Alumina Co., Ltd., Liaocheng, Shandong Province, China. Blast furnace slag (BFS) was provided by Hebei Jingye Steel Mill, Shijiazhuang, China. Calcium compounds of CaO, CaCl_2_, and CaCO_3_ (AR) and sodium silicate solution (8.3 wt% Na_2_O, 26.5 wt% SiO_2_, 65.2 wt% H_2_O) were provided by the Aladdin Chemical Reagents (Shanghai, China). The sodium hydroxide solid pellets (AR) were used for adjusting the modulus of the activators. Distilled water was used in the tests. The chemical compositions of RM and BFS were analyzed using X-ray fluorescence spectrometry (XRF) (PANalytical Axios, Malvern, UK), as shown in Table 1. The main components of RM were Fe_2_O_3_ (46.19%), Al_2_O_3_ (22.4%), and SiO_2_ (13.73%), while the BFS contains CaO (42.58%) besides SiO_2_ (28.84%) and Al_2_O_3_ (14.46%). The XRD results of RM and BFS are shown in Figure 1. The crystalline phases in RM were mainly sodalite (Na_8_(Al_6_Si_6_O_24_)(OH)_2_), gibbsite (Al(OH)_3_), goethite (FeO(OH)), hematite (Fe_2_O_3_), and quartz (SiO_2_). Of these, gibbsite (Al(OH)_3_) is considered to be the active ingredient in RM. The BFS showed several low-intensity peaks that were related to calcium silicate (Ca_8_Si_5_O_13_) and calcite (CaCO_3_), with a broad amorphous hump between about 25° and 35°.

### 2.2. Method

Based on previous studies and works, a formulation with a 1:1 mass ratio of RM to BFS was selected as the raw material in this study. Table 2 gave the parameters for synthesizing RM-BFS-based geopolymer with various ratios of calcium compounds. The dosage of calcium compounds was calculated according to the mass fraction of the raw material, with the dosage of CaO and CaCO_3_ incrementally increasing from 1, 2, and 3 to 4% and the dosage of CaCl_2_ incrementally increasing from 0.5, 1, and 1.5 to 2%. The maximum dosage was based on the dosage of paste that could be molded after mixing was completed. In addition, a control group without any calcium compounds was prepared as a reference. The alkaline activators were homogenous solutions mixed with sodium silicate solution, sodium hydroxide pellets, and distilled water, while the modulus of the activators was adjusted from 3.3 to 1.5 using sodium hydroxide solid pellets, which had been prepared and sealed at room conditions of 25.0 ± 2.0 °C and a relative humidity of 50 ± 10% for more than 12 h before preparation. The ratio of alkaline activators to the mass of RM and BFS was 0.25. The ratio of water to mass of RM and BFS was 0.38, where water includes both the water in the sodium silicate solution and the additional water added.

For the preparation of the geopolymer samples, the dried RM and BFS powders were first mixed using a blender. The activator was stirred slowly, then half of the powders were added to the activator and stirred slowly for 2 min, and the remaining powders were added to the paste and stirred rapidly for 2 min. It should be mentioned that for the rapid-hardening samples, we reduced the mixing time appropriately for better molding. When the mixing was completed, the freshly mixed paste was immediately tested for setting time and fluidity, and the remaining paste was poured into a steel mold (30 mm × 30 mm × 30 mm). After that, the molds were placed on a vibration table for 2 min to allow the samples to be fully incorporated into the molds, and the molds were wrapped with plastic film and placed under room conditions of 25.0 ± 2.0 °C and a relative humidity of 50 ± 10%. After 2 days, the samples were demolded and continued to be kept under the same room conditions until 7 and 28 days.

### 2.3. Measurements

The setting times of the geopolymers were determined through a Vicat needle test, according to the Chinese standard (GB/T 1346-2011) [21]. When the mixing of the geopolymer was completed, the paste was cast into a truncated cone mold with dimensions of φ65 mm × φ75 mm × 40 mm, and the upper surface was scraped. The initial setting time was defined as the time that the addition of powder to the activator until the needle was inserted into the paste 4 ± 1 mm from the bottom of the mold. After the initial setting time determination, the mold was turned over with the paste for the final setting time determination step immediately. The final setting time was determined as the needle sank into the paste by no more than 0.5 mm. The results of the setting time were obtained by taking the average of three samples.

The flowability of the fresh paste was measured regarding the test method for the fluidity of cement mortar according to the Chinese standard (GB/T 8077-2012) [22]. When the mixing of geopolymer was completed, the paste was cast into a truncated cone mold with dimensions of φ36 mm × φ60 mm × 60 mm placed on a glass plate. Then, the mold was lifted in a vertical direction so that the paste flowed on the glass plate. After 30 s, the maximum diameter of the flowing portion of the flow in both directions perpendicular was measured, which was averaged to take the flowability of the paste. We repeated the process three times to evaluate the result.

The chemical composition and phases of RM, BFS and geopolymers were characterized using X-ray diffractometry with Cu-Ka radiation (DY1602, Empyrean, Hong Kong, China), employing 2θ values of 5–90°. The compressive strengths of the geopolymers were tested using a compression and flexure machine (DNS100, Jinan Tianchen Co., Ltd., Jinan, China). The results of the compressive strength were evaluated according to three samples. The surfaces of geopolymers were analyzed by using XPS (ESCALAB 250, Thermo Scientific, Waltham, MA, USA). The heat evolution of the geopolymers was tested within 30 h using a micro-calorimeter (TAM Air, Tbilisi, Georgia) to investigate the differences in the process of geopolymerization reaction between different calcium species. The SEM (Quanta 250, Fremont, CA, USA) was selected to characterize the microstructure of RM-BFS-based geopolymers with different calcium species.

## 3. Results

### 3.1. Mechanical Strength Analysis

**Figure 2:**
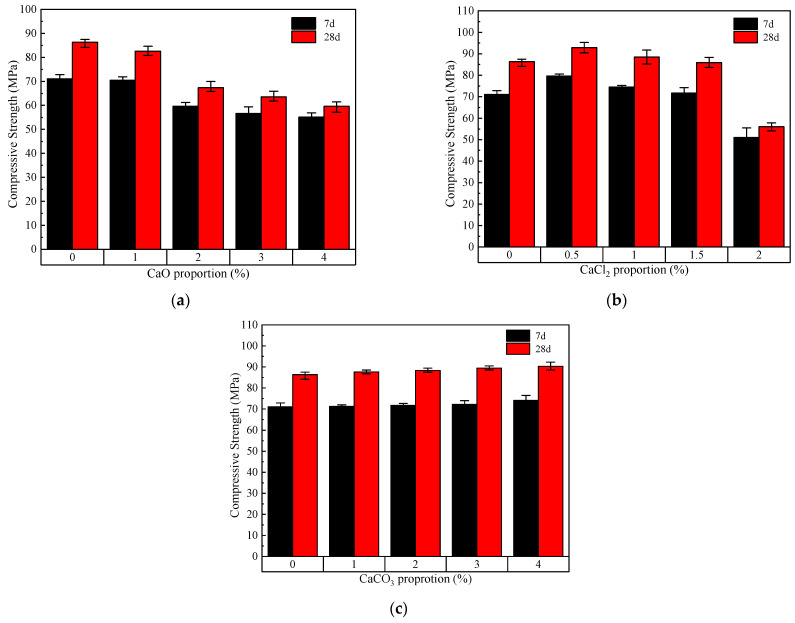
Compressive strengths of RM-BFS-based geopolymers with various calcium compounds: (**a**) geopolymers with CaO; (**b**) geopolymers with CaCl_2_; (**c**) geopolymers with CaCO_3_.

The 7-day and 28-day compressive strengths of RM-BFS-based geopolymers with different calcium species are shown in Figure 2. With the increase in the CaO proportion from 0% to 4%, the compressive strengths of the geopolymers at 7 days and 28 days decreased from 71.1 MPa and 86.3 MPa to 55.1 MPa and 59.6 MPa, respectively (Figure 2a). This is because, on one hand, CaO may accelerate the geopolymerization reaction to shorten the setting time, but it cannot effectively construct a large-scale network structure; on the other hand, CaO will react off a large amount of water, especially if the CaO proportion is higher, while the heat released will cause the water to evaporate and form a large number of pores, making it difficult for it to be filled by the condensed geopolymers. Accordingly, the compressive strengths of the geopolymers at 7 days and 28 days showed a trend of increasing and then decreasing when CaCl_2_ was added. The compressive strengths of the geopolymers at 7 days and 28 days were highest when the CaCl_2_ proportion was 0.5%, being 79.7 MPa and 92.9 MPa, respectively (Figure 2b). This indicated that a proportion of 0.5% CaCl_2_ was optimal in terms of compressive strength. It was possible that a gel structure was generated when an appropriate proportion of CaCl_2_ was added, which wrapped the unreacted particles as aggregates to fill in the pores formed by water evaporation. However, the filling effect of the aggregates formed by the gels and the particles was not fully reflected when the CaCl_2_ was excessive because there was no stable structure constructed in the geopolymer. As shown in Figure 2c, the compressive strength of the geopolymers did not change significantly with the increase in CaCO_3_ proportion from 0% to 4%, which further indicates that the CaCO_3_ particles played the role of filling fine aggregate in the paste and slightly increased the compressive strength.

### 3.2. Setting Time and Fluidity Analysis

**Figure 3:**
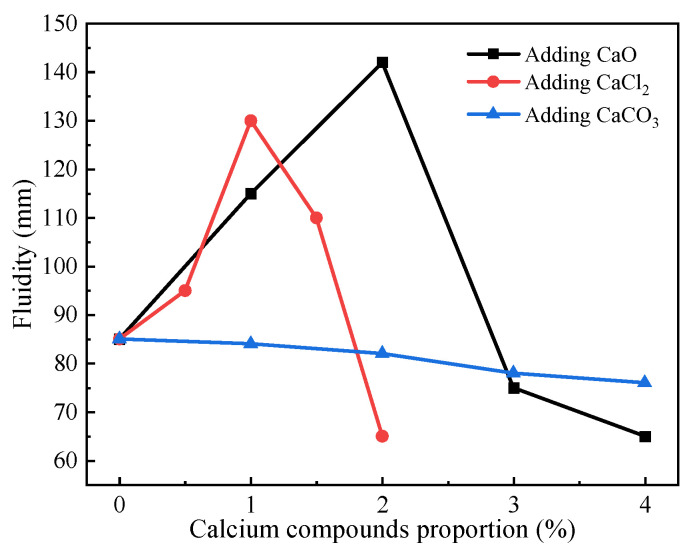
The fluidity of RM-BFS-based geopolymers with different calcium species.

The fluidity results of the geopolymer paste are shown in Figure 3. Fluidity is the ability of pastes to flow freely for a certain period. Pastes with low fluidity are not favorable for practical applications. Figure 3 indicated that the flowability of geopolymer paste showed a trend of increasing and then decreasing with the increases in CaO and CaCl_2_ proportions. The fluidity of the paste was maximum when the CaO and CaCl_2_ proportions were 2% and 1%, respectively, which were 142 mm and 130 mm. This may be because moderate amounts of calcium compounds were involved in the reaction during mixing, which increased the temperature of the paste and slightly reduced the viscosity of the paste, resulting in an increase in the fluidity. But, the paste hardly flowed because of rapid condensation when the CaO and CaCl_2_ proportions were 4% and 2%, respectively. It was seen that the fluidity of the paste decreased slightly as the CaCO_3_ proportion increased from 0% to 4%.

**Figure 4:**
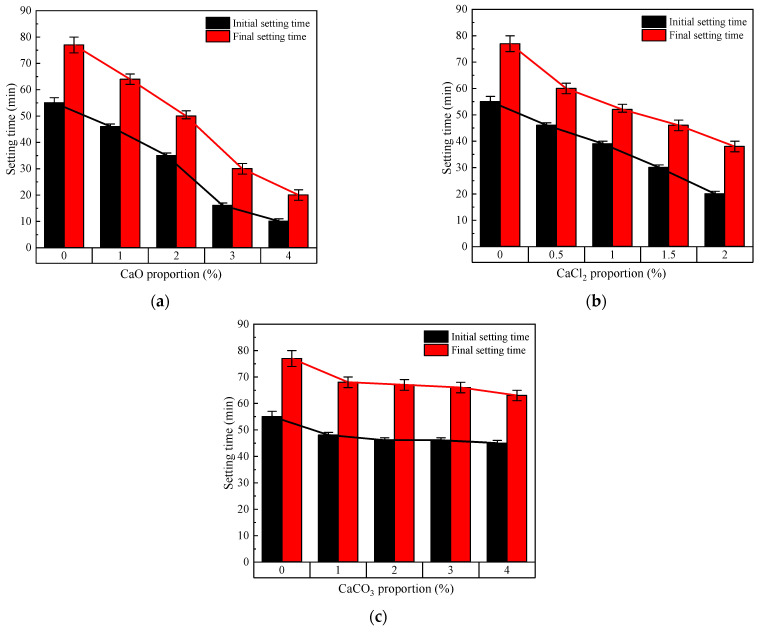
The setting times of RM-BFS-based geopolymers with different calcium species: (**a**) geopolymers with CaO; (**b**) geopolymers with CaCl_2_; (**c**) geopolymers with CaCO_3_.

The setting time test results of the geopolymer paste are shown in Figure 4. With the increase in CaO proportion, not only were the initial and final setting times of the geopolymer shortened, but so was the difference between the initial and final setting time, as shown in Figure 4a. The effect of CaCl_2_ on the setting time of the geopolymer paste was similar to that of CaO, but the proportion corresponding to CaCl_2_ was significantly lower. Combined with Figure 3 and Figure 4c, insoluble CaCO_3_ had no significant effect on the setting time of the paste when the proportion was less than 5%. Therefore, it could be assumed that the CaCO_3_ acted as a fine aggregate for filling the paste, thus slightly reducing the fluidity of the paste.

### 3.3. XRD Analysis

**Figure 5:**
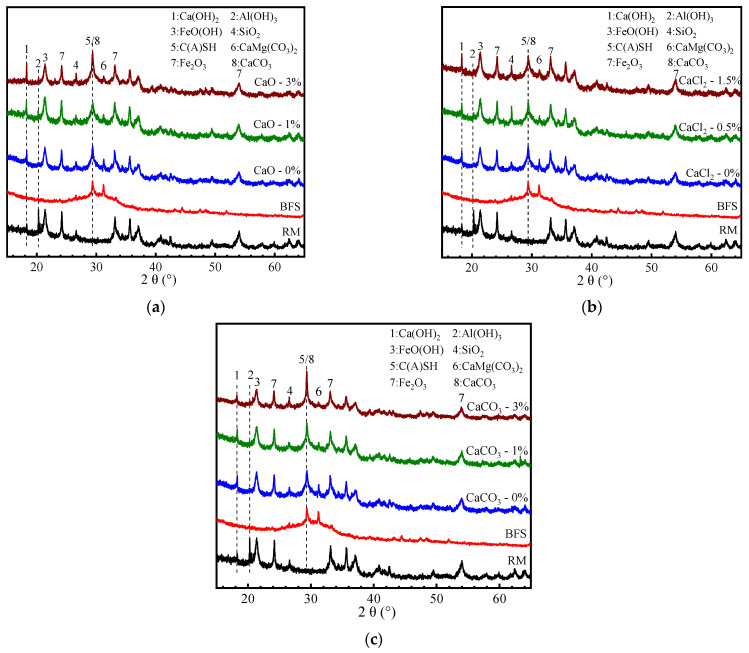
XRD patterns of RM-BFS-based geopolymers with different calcium species: (**a**) geopolymers with CaO; (**b**) geopolymers with CaCl_2_; (**c**) geopolymers with CaCO_3_.

Figure 5 shows the XRD patterns of RM-BFS-based geopolymers with the addition of CaO and CaCO_3_, from 0% and 1% to 3%, and CaCl_2_, from 0% and 0.5% to 1.5%, for comparison with the XRD patterns of RM and BFS. In all the XRD patterns, the diffraction peak of gibbsite (Al(OH)_3_) at 20.3° was found to have disappeared, which indicated that the gibbsite in RM was dissolved under alkaline conditions. The crystalline phases produced by RM, such as quartz (SiO_2_), goethite (FeO(OH)), and hematite (Fe_2_O_3_), with diffraction peaks of 26.6°, 21.2°, and 33.2°, respectively, were still present in all the XRD patterns, suggesting that these crystalline phases in the RM were inert in alkaline conditions [23]. The diffraction peaks at 29.4° were related to calcite (CaCO_3_), which was possibly attributed to production by the reaction of Ca(OH)_2_ and CO_2_ in the air. According to the XRD patterns, the diffraction peaks located at 28–32° corresponded to C-(A)-S-H gels [24]. Therefore, it is possible that some N(C)-A-S-H gels were formed, but these gels were difficult to characterize by XRD analysis due to their amorphous or nanocrystalline natures [25]. As shown in Figure 5a, the diffraction peak of Ca(OH)_2_ located at 18.2° appeared, indicating that the addition of CaO generated Ca(OH)_2_. Similarly, it can be seen in geopolymers with CaCl_2_ added (Figure 5b). Nucleation is one of the most important steps in the geopolymerization reaction, referring to the development of aluminosilicate oligomers into aluminosilicate quasi- or nanocrystalline [26]. Combined with Figure 4 and Figure 5, it is believed that Ca(OH)_2_ generated immediately precipitated crystals, due to its low solubility in alkaline conditions, when CaO and CaCl_2_ were added, which provided potential nucleation sites at the solid–liquid boundaries in the initial stages of geopolymerization [26]. The diffraction peak of calcite (CaCO_3_) at 29.4° still existed regardless of the addition of CaCO_3_ (Figure 5c). This is the same as the view obtained from Figure 3 and Figure 4, showing that CaCO_3_ played the role of filling in the form of fine aggregate.

### 3.4. XPS Analysis

XPS is an important and powerful tool for studying the chemical states of elements. However, the technique has not been widely applied to the analysis of aluminosilicate networks [27]. Geopolymers and their raw materials contain a wide range of elements in different phases, which results in broad photoelectron spectra that are difficult to analyze. However, the O 1s photoelectron spectra play an important role in the geopolymerization process and can still be very helpful for extracting meaningful information [28].

**Figure 6:**
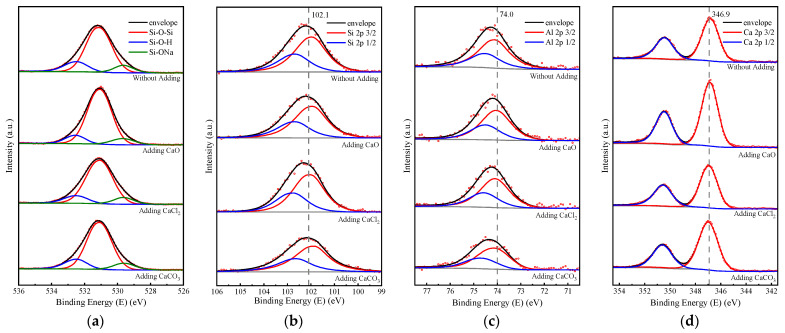
Fitted XPS spectra for the RM-BFS-based geopolymer with different calcium species: (**a**) O 1s; (**b**) Si 2p; (**c**) Al 2p; (**d**) Ca 2p.

The XPS peaks and fitting results of geopolymer samples with different calcium compounds are shown in Figure 6, where geopolymer samples with 3% CaO and CaCO_3_ added and 1.5% CaCl_2_ added were chosen for testing. Table 3 summarizes the integral fitting results for O, Si, Al, and Ca atoms for each sample. In the study investigated by Kljajević [29], the O 1s line was decomposed into three contributions: the first peak was related to Si-ONa bonds located at about 529.5 eV, the second was related to the Si-O-Si bonds in silicate in the range of 531–532 eV [27], and the third (at about 533 eV) was related to Si-OH bonds [30]. In the meantime, the O 1s could also be divided into two types of sites, namely bridging sites, Br.O (Si-O-Si, 532.0 eV), and non-bridging sites, nBr.O (Si-OH, 533.0 eV and Si–ONa, 529.5 eV), because of the electrostatic interaction force [31].

Table 3 summarizes the contributions of the three different characteristic peaks. As pointed out by Kljajević [29], the ratio of c/a can roughly determine the degree of crosslinking of the geopolymer network, and a decrease in the c/a ratio indicates an increase in the degree of crosslinking of the geopolymer chains. When CaO and CaCl_2_ were added, the c/a ratio of the samples was reduced by a factor of 1.55 and 1.34. It is evident that the degree of interlocking of geopolymer chains could be improved to varying degrees after doping with calcium compounds. The Si 2p3/2 line of the geopolymers with calcium compounds shifted compared with the control group, indicating a change in the chemical environment around the Si atom. The Al 2p3/2 line of the geopolymer has been reported to be present in the range of 74.5 ± 0.5 eV due to oxygen or hydroxyl groups around the aluminum atom [28]. As shown in Table 3, the Al 2p3/2 line for the control group was located at 74.2 eV, while the peak shifted to a lower energy of 74.0 eV for the samples with CaO and CaCl_2_ added. During the geopolymerization reaction, the ligand of the aluminum atoms changed from octahedral to tetrahedral, and tetrahedral-coordinated aluminum had a much lower binding energy compared to octahedral coordination [30], which suggests that the ligand of Al in the geopolymer samples was closer to tetrahedral coordination doping with CaO and CaCl_2_.

The Ca 2p3/2 lines in the geopolymer samples were all located around 346.9 eV (Figure 6d), and the Ca 2p3/2 lines in the geopolymer samples added with calcium compounds were shifted towards higher binding energies compared to the control group, with the lines of the geopolymers with the addition of CaCO_3_ being located at higher binding energies. While CaCO_3_ was added to the system as a single phase, both CaO and CaCl_2_ reacted in the system to form Ca(OH)_2_. This suggests that Ca(OH)_2_ generated by adding calcium compounds underwent different degrees of carbonation with CO_2_ in the air.

### 3.5. Heat Evolution Analysis

According to previous studies [32], for the hydration exothermic rate curve of a geopolymer, three exothermic peaks generally appear. The first exothermic peak is the wetting and dissolution of the raw material powder. The second exothermic peak refers to the reaction between the dissolved calcium ions of the raw material and the silicate ions in the solution, which originate from the activators. The third exothermic peak originates from the geopolymerization reaction. Of the three peaks above, the first and second exothermic processes usually take place simultaneously, so sometimes they may show a single exothermic peak. From the above results, it can be seen that the first two exothermic peaks of the geopolymers overlap and the reaction is rapid, mainly occurring within 3 h.

**Figure 7:**
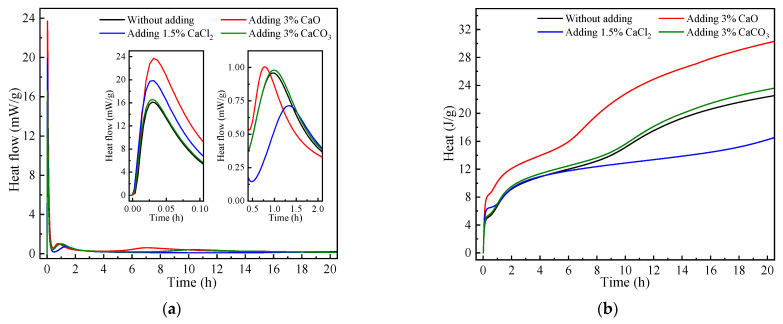
Heat evolution of RM-BFS-based geopolymers with different calcium species: (**a**) heat flow curves; (**b**) accumulative heat release curves.

As shown in Figure 7a, all geopolymer samples showed double peaks within 3 h of the reaction start, and continuous exotherm was found within 20 h of the reaction start. The initial exothermic peaks of the four groups of samples were formed by the overlap of two exothermic peaks, which were the dissolution of raw material powders and the reaction of dissolved Ca^2+^, while the second exothermic peaks all originated from the geopolymerization reaction. The initial exothermic peak was the highest among the four groups of samples when CaO was added, reaching 24 mW/g, which is due to the reaction between CaO and water that releases a large amount of heat. The intensity of the initial exothermic peak was second when CaCl_2_ was added, with an exothermic rate close to 20 mW/g. The difference with the control group was due to the dissolution of the CaCl_2_ particles and the reaction of dissolved Ca^2+^. After adding CaCO_3_, the intensity of the initial exothermic peak increased only a little compared with the control group, which represented the fact that in addition to the exothermic dissolution of the raw material, a minimal amount of Ca^2+^ reaction exotherm occurred. Such exothermic behavior was basically like that of the control group, which suggested the inertness of the CaCO_3_ again [33].

In Figure 7a, the peaks at 0.5–2 h correspond to the geopolymerization reaction. Compared with the geopolymer with CaO and CaCl_2_ added, XRD analysis revealed that both produce Ca(OH)_2_, but there was a difference in the exothermic behavior. The geopolymer with CaO added showed earlier, higher, and narrower peaks than the control group, while the geopolymer with CaCl_2_ added showed later, lower, and wider peaks than the control group. This may be due to the heat released to increase the temperature of the reaction system by the dissolution of CaO, which accelerated the geopolymerization reaction. As in [34], increasing the temperature was favorable for the reaction to proceed. Meanwhile, combined with the fact that CaCl_2_ can shorten the setting time of geopolymer, its mechanism of shortening the setting time was not manifested by accelerating the geopolymerization reaction. Therefore, it was presumed that for the mechanism of CaCl_2_ used to shorten the setting time of geopolymer, in addition to the dissolution of Ca^2+^ in alkaline solution under the generation of Ca(OH)_2_, Ca^2+^ dissolved from CaCl_2_ could also react with the SiO_3_^2−^ in the solution, causing the generation of CaSiO_3_ gel attached on the surfaces of particles, which we will discuss in detail in the SEM analysis. The cumulative heat of the geopolymer with CaCl_2_ within the first 1 h was greater than that of the control group, while the cumulative heat of the control group exceeded that of the geopolymer with CaCl_2_ after 1 h (Figure 7b), which once again suggested that the mechanism of shortening the setting time for CaCl_2_ was different from that for CaO, which was exothermic to promote geopolymerization reaction.

According to the results of compressive strength (Figure 2) and heat evolution (Figure 7) tests, the geopolymerization reaction process was completed in less than 2 h when the CaO proportion was 3%, and the compressive strengths at 7 d and 28 d were lower than in the control group, being 56.6 MPa and 63.5 MPa and 71.1 MPa and 86.3 MPa, respectively. The compressive strengths of the geopolymers with a 1.5% proportion of CaCl_2_ did not change significantly and were 71.7 MPa and 85.9 MPa, respectively. This indicated that shortening the setting time of the paste by increasing the temperature of the reaction system was likely to have a certain negative impact on the compressive strengths of the geopolymers. On the other hand, it confirmed that the addition of CaCl_2_ has a different mechanism of shortening the setting time from that of CaO.

### 3.6. SEM Analysis

**Figure 8:**
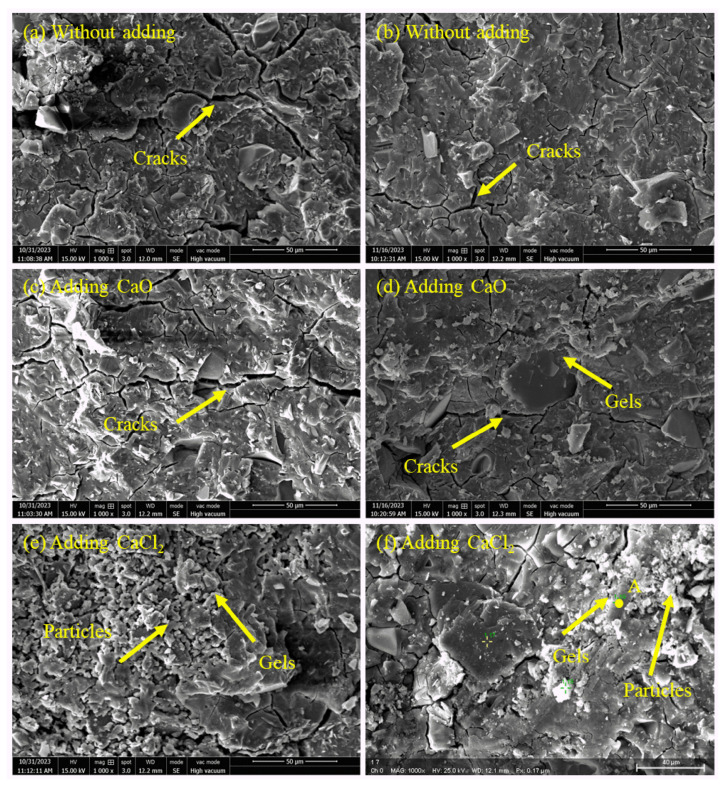
SEM images of the RM-BFS-based geopolymer with different calcium species: (**a**,**b**) geopolymers without adding, (**c**,**d**) geopolymers with CaO; (**e**,**f**) geopolymers with CaCl_2_.

Figure 8 shows the SEM images of the RM-BFS-based geopolymer added with 3% CaO, the geopolymer added with 1.5% CaCl_2_, and the control group. In the SEM images of the control group (Figure 8a,b), some microcracks were found and the surface structure of the geopolymer was relatively dense. In Figure 8c,d, many microcracks were observed, and the number and lengths of these microcracks were larger than those of the control group. Here, the formation of microcracks was mainly attributed to autogenous and drying shrinkage during the curing process. These cracks were likely to be caused by the thermal stresses generated by the exothermic reaction between CaO and water, which is consistent with previous assumptions. The addition of CaCl_2_ to the geopolymer formed a denser structure, and the number and lengths of microcracks were smaller than those of the control group (Figure 8e). The reason is that calcium ions dissolved by CaCl_2_ reacted with silicate ions to generate a hydrated calcium silicate gel, which filled the pores and cracks in the geopolymer. Hence, the compressive strength of the geopolymer with 1.5% CaCl_2_ was higher than that of the control group. It was also observed that some of the unreacted particles were encapsulated by the gel (Figure 8f), which confirmed the fact that the addition of CaCl_2_ delayed the exothermic peak of the geopolymerization reaction in the heat evolution analysis. EDS analysis showed that the main components of point A in Figure 8f were O (54.67%), Ca (7.70%), Si (12.54%), Na (14.88%) and Al (2.68%), suggesting that calcium hydrated silicate may be present and coexist with some of the N-A-S-H gels, which confirmed previous assumptions.

## 4. Conclusions

1. Other than calcium carbonate, both calcium oxide and calcium chloride played roles in accelerating the setting times of RM-BFS-based geopolymers. The increases in the proportions of CaO and CaCl_2_ resulted in reductions in both the initial and final setting times of the geopolymers, but the proportion corresponding to CaCl_2_ was significantly lower.
2. The proper addition of CaCl_2_ could improve the compressive strength of the RM-BFS-based geopolymer. When the CaCl_2_ content was 0.5%, the compressive strengths of the geopolymer samples at 7 days and 28 days were the highest, being 79.7 MPa and 92.9 MPa, respectively. However, the excessiveness caused a decrease in the strength due to rapid hardening. The compressive strength of the geopolymer samples decreased with the increase in the CaO proportion. CaCO_3_ only acted as a fine aggregate for filling the paste.
3. The calcium compounds had different mechanisms to accelerate the setting times of RM-BFS-based geopolymers. CaO reacted with water to release massive heat to advance the geopolymerization and generate calcium hydroxide to provide nucleation sites for geopolymerization. CaCl_2_ had a common mechanism for providing nucleation sites for the geopolymerization, as well as the generation, of hydrated calcium silicate gel, which could fill the pores generated by water evaporation. Building upon the findings of this study, a distinct opportunity exists for further research, particularly to investigate the underlying mechanisms by which calcium ions influence the geopolymerization reaction, with a focus on the formation and structural characteristics of geopolymer gels.

## Figures and Tables

**Table 1:** The chemical compositions (wt%) of the RM and BFS.

Components (%)	SiO_2_	Al_2_O_3_	Na_2_O	CaO	Fe_2_O_3_	TiO_2_	K_2_O	MgO	SO_3_
RM	13.73	22.4	10.13	0.61	46.19	5.51	0.17	0.12	0.65
BFS	28.84	14.46	0.46	42.58	0.57	1.39	0.41	7.74	2.75

**Table 2:** Preparation of RM-BFS-based geopolymer.

No.	Raw Materials					Alkaline Activator/g	Water/g
	RM/g	BFS/g	CaO/g	CaCl_2_/g	CaCO_3_/g		
0	250	250	-	-	-	125	114.6
1	250	250	5	-	-	125	114.6
2	250	250	10	-	-	125	114.6
3	250	250	15	-	-	125	114.6
4	250	250	20	-	-	125	114.6
5	250	250	-	2.5	-	125	114.6
6	250	250	-	5	-	125	114.6
7	250	250	-	7.5	-	125	114.6
8	250	250	-	10	-	125	114.6
9	250	250	-	-	5	125	114.6
10	250	250	-	-	10	125	114.6
11	250	250	-	-	15	125	114.6
12	250	250	-	-	20	125	114.6

**Table 3:** Fitted results of the O 1s, Si 2p, Al 2p, and Ca 2p lines taken from geopolymer samples with different calcium species.

Sample	O 1s (eV/%)			Si 2p3/2(eV)	Al 2p3/2(eV)	Ca 2p3/2(eV)
	a	b	c			
Without Adding	531.1/70.38	532.5/18.01	529.6/11.61	101.8	74.2	346.8
Adding CaO	531.1/79.75	532.6/11.74	529.7/8.51	101.9	74.0	346.9
Adding CaCl_2_	531.1/77.37	532.5/13.09	529.6/9.54	102.1	74.0	346.9
Adding CaCO_3_	531.1/71.43	532.5/17.93	529.6/10.64	101.8	74.1	347.0

## Data Availability

The original contributions presented in the study are included in the article, further inquiries can be directed to the corresponding author.

## References

[1] Lazorenko G., Kasprzhitskii A. (2022). Geopolymer additive manufacturing: A review. Addit. Manuf..

[2] Chen K.Y., Wu D.Z., Xia L.L., Cai Q.M., Zhang Z.Y. (2021). Geopolymer concrete durability subjected to aggressive environments—A review of influence factors and comparison with ordinary Portland cement. Constr. Build. Mater..

[3] Lei M.K., Wang X., Meng H., Yan Z.X., Lin J.H., Wu Z.S. (2023). Study of fly ash-slag geopolymer mortar as a rapid strengthening agent for concrete structures. Constr. Build. Mater..

[4] Adufu Y.D., Sore S.O., Nshimiyimana P., Messan A., Escadeillas G. (2023). Effect of curing conditions on physico-mechanical properties of metakaolin-based geopolymer concrete containing calcium carbide residue. Mrs Adv..

[5] Saloni, Singh A., Sandhu V., Jatin P. (2020). In Effects of alccofine and curing conditions on properties of low calcium fly ash-based geopolymer concrete. Mater. Today Proc..

[6] Temuujin J., van Riessen A., Williams R. (2009). Influence of calcium compounds on the mechanical properties of fly ash geopolymer pastes. J. Hazard. Mater..

[7] Yip C.K., Lukey G.C., Provis J.L., van Deventer J.S.J. (2008). Effect of calcium silicate sources on geopolymerisation. Cem. Concr. Res..

[8] Bahmani H., Mostofinejad D. (2023). High-performance concrete based on alkaline earth metal ions-activated slag at ambient temperature: Mechanical and microstructure properties. J. Mater. Res. Technol..

[9] Zhang Z.H., Wang H., Zhu Y.C., Reid A., Provis J.L., Bullen F. (2014). Using fly ash to partially substitute metakaolin in geopolymer synthesis. Appl. Clay Sci..

[10] Shang J., Dai J.G., Zhao T.J., Guo S.Y., Zhang P., Mu B. (2018). Alternation of traditional cement mortars using fly ash-based geopolymer mortars modified by slag. J. Clean. Prod..

[11] Arnoult M., Perronnet M., Autef A., Rossignol S. (2018). How to control the geopolymer setting time with the alkaline silicate solution. J. Non-Cryst. Solids.

[12] Nath P., Sarker P.K. (2014). Effect of GGBFS on setting, workability and early strength properties of fly ash geopolymer concrete cured in ambient condition. Constr. Build. Mater..

[13] Hadi M.N.S., Zhang H.Q., Parkinson S. (2019). Optimum mix design of geopolymer pastes and concretes cured in ambient condition based on compressive strength, setting time and workability. J. Build. Eng..

[14] Tian K.G., Wang Y.S., Dong B.Q., Fang G.H., Xing F. (2022). Engineering and micro-properties of alkali-activated slag pastes with Bayer red mud. Constr. Build. Mater..

[15] Xie J.H., Chen W., Wang J.J., Fang C., Zhang B.X., Liu F. (2019). Coupling effects of recycled aggregate and GGBS/metakaolin on physicochemical properties of geopolymer concrete. Constr. Build. Mater..

[16] Mucsi G., Szabó R., Rácz A., Kristály F., Kumar S. (2019). Combined utilization of red mud and mechanically activated fly ash in geopolymers. Rud.-Geol.-Naft. Zb..

[17] Kumar A., Saravanan T.J., Bisht K., Kabeer K. (2021). A review on the utilization of red mud for the production of geopolymer and alkali activated concrete. Constr. Build. Mater..

[18] Zhang J., Li S.C., Li Z.F., Gao Y.F., Liu C., Qi Y.H. (2021). Workability and microstructural properties of red-mud-based geopolymer with different particle sizes. Adv. Cem. Res..

[19] Ahmed S., Meng T., Taha M. (2020). Utilization of red mud for producing a high strength binder by composition optimization and nano strengthening. Nanotechnol. Rev..

[20] Albidah A.S. (2021). Effect of partial replacement of geopolymer binder materials on the fresh and mechanical properties: A review. Ceram. Int..

[21] (2011). Test Methods for Water Requirement of Normal Consistency, Setting Time and Soundness of the Portland Cement.

[22] (2012). Methods for Testing Uniformity of Concrete Admixture.

[23] Lemougna P.N., Wang K.T., Tang Q., Cui X.M. (2017). Study on the development of inorganic polymers from red mud and slag system: Application in mortar and lightweight materials. Constr. Build. Mater..

[24] Wang Y.G., Liu X.M., Tang B.W., Li Y., Zhang W., Xue Y. (2021). Effect of Ca/(Si plus Al) on red mud based eco-friendly revetment block: Microstructure, durability and environmental performance. Constr. Build. Mater..

[25] Gong C.M., Yang N.R. (2000). Effect of phosphate on the hydration of alkali-activated red mud-slag cementitious material. Cem. Concr. Res..

[26] van Deventer J.S.J., Provis J.L., Duxson P., Lukey G.C. (2007). Reaction mechanisms in the geopolymeric conversion of inorganic waste to useful products. J. Hazard. Mater..

[27] Simonsen M.E., Sonderby C., Li Z.S., Sogaard E.G. (2009). XPS and FT-IR investigation of silicate polymers. J. Mater. Sci..

[28] Mudgal M., Singh A., Chouhan R.K. (2022). Fly ash red mud geopolymer with improved mechanical strength. Clean. Eng. Technol..

[29] Kljajevic L.M., Nenadovic S.S., Nenadovic M.T., Bundaleski N.K., Todorovic B.Z., Pavlovic V.B., Rakocevic Z.L. (2017). Structural and chemical properties of thermally treated geopolymer samples. Ceram. Int..

[30] Kanuchova M., Kozakova L., Drabova M., Sisol M., Estokova A., Kanuch J., Skvarla J. (2015). Monitoring and characterization of creation of geopolymers prepared from fly ash and metakaolin by X-ray photoelectron spectroscopy method. Environ. Prog. Sustain. Energy.

[31] Revathi T., Jeyalakshmi R. (2019). XPS, 29Si, 27Al, 11B MAS-NMR, ATR-IR and FESEM characterization of geopolymer based on borax modified water glass activated Fly ash-GGBS blend. Mater. Res. Express.

[32] Granizo M.L., Alonso S., Blanco-Varela M.T., Palomo A. (2002). Alkaline activation of metakaolin: Effect of calcium hydroxide in the products of reaction. J. Am. Ceram. Soc..

[33] Yip C.K., Provis J.L., Lukey G.C., van Deventer J.S.J. (2008). Carbonate mineral addition to metakaolin-based geopolymers. Cem. Concr. Compos..

[34] Cai J.M., Li X.P., Tan J.W., Vandevyvere B. (2020). Thermal and compressive behaviors of fly ash and metakaolin-based geopolymer. J. Build. Eng..

